# To Embed or Not: Network Embedding as a Paradigm in Computational Biology

**DOI:** 10.3389/fgene.2019.00381

**Published:** 2019-05-01

**Authors:** Walter Nelson, Marinka Zitnik, Bo Wang, Jure Leskovec, Anna Goldenberg, Roded Sharan

**Affiliations:** ^1^Genetics and Genome Biology, SickKids Research Institute, Toronto, ON, Canada; ^2^Department of Cell and Systems Biology, University of Toronto, Toronto, ON, Canada; ^3^Department of Computer Science, Stanford University, Stanford, CA, United States; ^4^Peter Munk Cardiac Center, University Health Network, Toronto, ON, Canada; ^5^Vector Institute, Toronto, ON, Canada; ^6^Chan Zuckerberg Biohub, San Francisco, CA, United States; ^7^Department of Computer Science, University of Toronto, Toronto, ON, Canada; ^8^School of Computer Science, Tel Aviv University, Tel Aviv, Israel

**Keywords:** network biology, network embedding, network alignment, community detection, protein function prediction

## Abstract

Current technology is producing high throughput biomedical data at an ever-growing rate. A common approach to interpreting such data is through network-based analyses. Since biological networks are notoriously complex and hard to decipher, a growing body of work applies graph embedding techniques to simplify, visualize, and facilitate the analysis of the resulting networks. In this review, we survey traditional and new approaches for graph embedding and compare their application to fundamental problems in network biology with using the networks directly. We consider a broad variety of applications including protein network alignment, community detection, and protein function prediction. We find that in all of these domains both types of approaches are of value and their performance depends on the evaluation measures being used and the goal of the project. In particular, network embedding methods outshine direct methods according to some of those measures and are, thus, an essential tool in bioinformatics research.

## Introduction

Network biology is a powerful paradigm for representing, interpreting and visualizing biological data (Barabási and Oltvai, 2004). One of the standard approaches to computing on networks is to transform such data into vectorial data, aka *network embedding*, to facilitate similarity search, clustering and visualization (Hamilton et al., 2017b; Cai et al., 2018).

In a network embedding problem, one is given a network and an induced similarity (or distance) function between its nodes; the goal is to find a low dimensional representation of the network nodes in some metric space so that the given similarity (or distance) function is preserved as much as possible. For example, if the input network is unweighted and the distance between nodes is defined to be the graph geodesic distance, then a possible goal could be to find an embedding into Euclidean space that minimizes the sum of squared differences between graph distances and the corresponding Euclidean distances (Tenenbaum, 2000).

The classical approach to network embedding employs matrix factorization and is based on the fact that if the desired similarity matrix is positive semi-definite then it can be decomposed into the product of a real matrix and its transpose. Thus, if one represents each node by a row of that matrix then the given similarity is completely captured by the dot-product between the corresponding vector representations. Similarly, if one is given distances between nodes that satisfy the triangle inequality then double centering the distance matrix gives a positive semi-definite matrix whose decomposition yields vector representations that respect the given distances. This approach is precisely the multidimensional scaling procedure (Cox and Cox, 2000).

Embedding approaches have several potential advantages. Algorithms making use of embeddings are frequently faster than their counterparts which operate on the original networks. Additionally, the learned embeddings are often applicable for downstream analysis, either by direct interpretation of the embedding space or through the application of machine learning techniques which are designed for vectorial data. Beyond its computational advantages, network embedding is natural to use in biological problems that concern physical entities (such as proteins) that function in 3D space. In such scenarios, Euclidean representations may capture many of the functional properties of those entities. Finally, by working in lower dimensional space, the results are more likely to be robust to the noise inherently present in the networks. Indeed, recent network denoising approaches employed embedding for this purpose (Wang et al., 2018).

**FIGURE 1:**
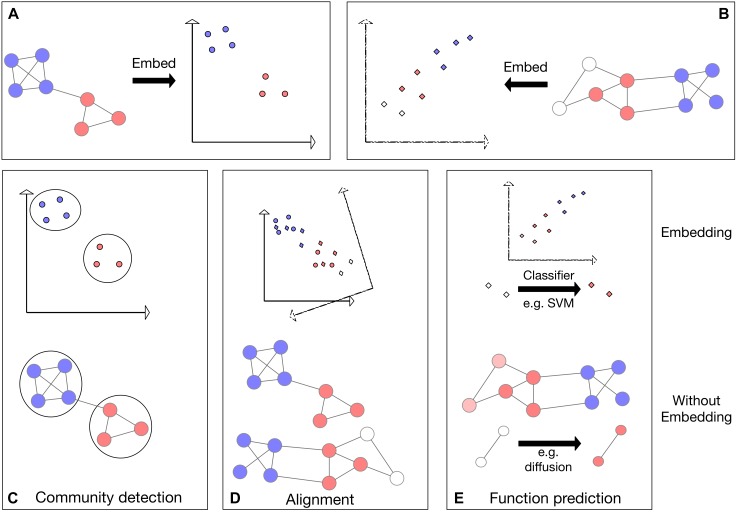
Schematic representing three applications applied to networks directly as well as applied to the network embeddings. Colors represent some node features in the network; for example, protein families. **(A,B)** Visualization of the embedding process for two networks in 2D space. **(C)** Visualization of community detection in embedded space (*top*) and directly on the network (*bottom*). **(D)** *Top*: visualization of network alignment in embedded space. In this example, the network embedding in panel **(B)** is rotated, translated and reflected to find an optimal alignment with the embedding in panel **(A)**. *Bottom*: visualization of direct alignment of two networks: vertical proximity represents the found alignment. **(E)** Visualization of function prediction in embedded space. The previously unlabeled (white) nodes (*bottom*) or their embeddings (*top*) are labeled (colored).

In this review, we describe several current approaches for graph embedding including spectral-based, diffusion-based and deep-learning-based methods. We provide comparisons applying representative embedding approaches to fundamental problems in network biology with using the networks directly in three distinct tasks: protein network alignment, protein module detection, and protein function prediction (Figure 1). We further review network embedding methods and their application to network denoising and pharmacogenomics. We conclude that network embedding methods are an essential component in the bioinformatics tool box.

## Methodology

Methods for network embedding aim to optimize the difference between the node similarities/distances in the original network space and their similarities/distances under the embedding, which is typically constrained to have a low dimension. In the following, we describe various methods for embedding a given network in Euclidean space. For a graph *G* with *n* nodes, a weighted adjacency matrix *W* and a diagonal degree matrix *D*, we define its Laplacian matrix as *L = D-W*.

Graph drawing algorithms are perhaps the best-known embedding techniques, commonly used to visualize a graph in 2D space. Initially proposed in (Eades, 1984) as an extension of (Tutte, 1963), and further developed in (Fruchterman and Reingold, 1991), the spring-embedder model is a particularly elegant example: one can imagine that connected pairs of nodes are attached to springs which bring them closer together, while all nodes repel each other so as not to be placed too closely together. Other classes of graph drawing algorithms, including multi-level and dimensionality reduction-based techniques, are described in detail in a recent review (Gibson et al., 2013). Spatial analysis of functional enrichment (Baryshnikova, 2018) is one recent application of force-directed graph drawing algorithm, designed for the annotation and visualization of large, complex biological networks.

One of the fundamental methods to decompose a matrix is spectral decomposition, i.e., decomposing the matrix into its eigenvectors and eigenvalues. Given a network, the principal eigenvectors *Q* of its Laplacian matrix capture membership of nodes in implicit network clusters, commonly used for embedding (Belkin and Niyogi, 2003). The matrix *Q* is obtained by optimizing *min*_Q∈R^n×C^_*Trace*(*Q*^T^*L*^+^*Q*), s.t. *Q*^T^*Q* = *I*, where *L*^+^ = I-D^−1/2^*WD*^−1/2^ is a normalized Laplacian and *C* is the number of clusters. However, this spectral embedding reflects the global structure in the network without taking into consideration more fine-grained local structures and is therefore sensitive to noise. Wang et al. (2017a) recently introduced the Vicus matrix as a local-neighborhood version of the Laplacian matrix. Each cell of the Vicus matrix represents the probability of node *j* having the same label as node *i* if we did a random walk around the local neighborhood of node *i*. Encoding local neighborhoods in this fashion does not only preserve the geometric properties of the original Laplacian matrix but also reduces the noise and improves the quality of the embedding. Wang et al. showed that for a variety of tasks, including network clustering of single-cell RNA-seq data, cluster stability, identification of rare cell populations, and ranking of genes associated with cancer subtypes, Vicus-based spectral methods outperformed Laplacian-based spectral methods on a wide variety of biological tasks.

Diffusion-based approaches focus on embedding nodes into low-dimensional vector spaces by first using random walks to construct a network neighborhood of every node in the network, and then optimizing an objective function with network neighborhoods as input (Perozzi et al., 2014a; Tang et al., 2015; Grover and Leskovec, 2016). The objective function is carefully designed to preserve both the local and global network structures. For example, a popular method, Mashup, complements traditional random walks, which yield only diffusion states, with a dimensionality reduction step that is aimed at reducing the noise in these diffusion computations. To this end, Mashup approximates each diffusion state *s*_i_ with a multinomial logistic model based on a latent vector representation of nodes that uses far fewer dimensions than the original, *n*-dimensional state. Specifically, if the latent vector representation for node *i* is denoted by *x*_i_, Mashup also constructs a contextual vector *w*_i_ that has the same dimensionality as *x*_i_ and captures the topology of the subnetwork around node *i*. To this end, Mashup computes the probability assigned to node *j* in the diffusion state of node *i* as $\hat{s_{\text{ij}}}=\frac{\exp\left(x_{\text{i}}^{T}w_{\text{j}}\right)}{\sum_{\text{k}}\exp(x_{\text{i}}^{T}w_{\text{k}})}$, so that these computed diffusion states align with the original diffusion states. Mashup constructs an optimization framework to minimize the KL-divergence of these two diffusion states and applies standard gradient descent methods to solve for the latent representations.

Another widely used network embedding algorithm that uses random walks is node2vec (Grover and Leskovec, 2016). Node2vec learns node embeddings so that a node’s embedding can predict nearby (neighborhood) nodes. Technically, the network neighborhood *N(u)* is a set of nodes that appear in an appropriately biased, short random walk from node *u* (Grover and Leskovec, 2016). The goal of the algorithm is to find an embedding *f(u)* such that the conditional probability of observing *u*’s network neighbors *N(u)* is maximized. This conditional probability is modeled using a softmax function, leading to the following log likelihood: $\sum_{\text{u}}\sum_{v\in N(u)}\log\frac{\exp(f(u)\cdot f(v))}{\sum_{w}\exp(f(w)\cdot f(u))}$, across all nodes *u* in the network. Once embeddings are learned, one can use them for any downstream prediction task, including node classification, link prediction, and clustering. A similar network embedding algorithm is DeepWalk (Perozzi et al., 2014b). DeepWalk has been originally proposed to embed nodes in a social network setting, taking ideas from the linguistics literature (Perozzi et al., 2014b). In DeepWalk, the embeddings are learned based on truncated random walks which can be intuitively thought of as putting words (nodes) into sentences (sequences of nodes visited by a random walk). In the biological context, DeepWalk has been used to associate miRNAs with diseases (Li et al., 2017), predict drug target associations (Zong et al., 2017), and predict protein function (Kulmanov et al., 2017).

With the advent of deep learning methods, several deep learning approaches were proposed to embed networks. An important class of deep learning methods for network embedding are graph neural networks that generalize the notion of convolutions typically applied to image datasets to operations that can operate on arbitrary graphs (Defferrard et al., 2016; Kipf and Welling, 2016; Gilmer et al., 2017; Hamilton et al., 2017a). One can see graph neural networks as an embedding methodology that distills high-dimensional information about each node’s neighborhood into a dense vector embedding without requiring manual feature engineering (Defferrard et al., 2016; Kipf and Welling, 2016; Gilmer et al., 2017; Hamilton et al., 2017a). A graph neural network has two main components. First, the encoder, maps a node *u* to a low-dimensional embedding *f(u)*, based on *u*’s local neighborhood structure, its position in the graph, and/or its attributes. Next, the decoder takes the embeddings and extracts user-specified predictions from these embeddings. In contrast to embedding approaches that use random walks (reviewed above), graph neural networks support end-to-end learning. One can jointly optimize all trainable parameters and propagate gradients of the objective function through the encoder as well as the decoder. End-to-end learning can lead to substantial improvements in performance (Defferrard et al., 2016; Zitnik et al., 2018).

There has been significant recent interest in graph embeddings in non-Euclidean spaces. In particular, hyperbolic spaces have attracted much attention due to successful natural language processing models which use them for embedding words (Chamberlain et al., 2017). Muscoloni et al. (2017) describe a general algorithm termed “coalescent embedding” for embedding vertices in hyperbolic spaces. The algorithm proceeds by pre-weighting the network and applying a non-linear dimension reduction technique, followed by computing and adjusting the angular positions of the Euclidean embeddings and radial positioning according to node degree. More generally, networks and their respective embeddings can be interpreted geometrically, as described in recent reviews (Barthélemy, 2011; Papadopoulos et al., 2015; Moyano, 2017). These geometric models have been used successfully in applications to biological networks, particularly protein–protein interaction (PPI) networks (Serrano et al., 2012; Alanis-Lobato et al., 2016, 2018).

## Applications

### Network Alignment

A basic operation in biological research is to transfer knowledge across species. Indeed, sequence alignment has been the power horse of computational biology for almost five decades now. With the availability of physical interaction data, it was suggested to generalize alignment concepts to the network level (Kelley et al., 2003; Sharan and Ideker, 2006). There are several types of network alignment problems, here we focus on global network alignment where given the networks of two species (typically, PPI networks) one wishes to identify a 1–1 correspondence between the proteins of the two species under which the networks are most similar (Figure 1D).

A leading approach to this problem is the IsoRank algorithm (Singh et al., 2008) which is based on Google’s PageRank method, essentially measuring the correspondence, or similarity, between two proteins from different species based on the similarities of their neighboring nodes in the two corresponding networks. Thus, if we denote by *R*_ij_ the similarity between proteins *i* and *j* (from two different species), and we let *N(i)* denote the (open) neighborhood of protein *i* in its network, then:

$$R_{\text{ij}}=\frac{1}{\left|N(i)||N(j)\right|}\sum_{u\in N(i),v\in N(j)}R_{\mathrm{uv}}$$

These recursive equations give rise to an eigenvalue problem and their solution is used as an input to a maximum matching algorithm to compute the eventual correspondence.

Another, more recent approach is MAGNA (Saraph and Milenkoviæ, 2014) and its successor MAGNA++ (Vijayan et al., 2015). MAGNA uses a genetic algorithm to find the optimal alignment, where individuals are viewed as permutations of the nodes. Crossover relies on the notion of adjacency, where a pair of permutations is adjacent if they differ only by a single swap of two nodes; the crossover of two permutations is then the midpoint of the shortest path between the two permutations in the graph constructed from these adjacencies. Selection can be based on any metric, such as EC. MAGNA++ augments this approach by including cross-species node similarity information. An extensive review of methods for biological network alignment can be found in (Guzzi and Milenkovic, 2018) that mentions over thirty different approaches. Comparative network analysis methods are further reviewed in (Emmert-Streib et al., 2016).

A recent work by Fan et al. (2017) uses an embedding-based approach, MuNK, to compare networks across species by assessing similarity via embedded network topologies. The idea is to project the nodes of the two networks into the same Euclidean space in a way that preserves their intra-species network similarity and inter-species sequence similarity. For each species separately, a kernel similarity function is defined, and the corresponding embedding is computed by matrix decomposition. To tie the projections together, Fan et al. (2017) assume a given set of known matches, regarded as landmarks, between the two networks. A similar embedding approach that does not require a known subset of correspondences was suggested in (Heimann et al., 2018).

As a test case for network embedding, we evaluated the two algorithms, IsoRank and MuNK, using metrics of alignment quality. A common and simple metric is the edge correctness (EC), defined as the percentage of edges conserved under the mapping *f* (Kuchaiev et al., 2009; Clark and Kalita, 2014):

$$\mathrm{EC}=\frac{\left|f(E_{\text{A}})\cap E_{B}\right|}{\left|E_{\text{A}}\right|}\times 100\%$$

Note that the EC metric is asymmetric, and the order of the networks is traditionally chosen to maximize EC, i.e., *A* is chosen to be the smaller of the two networks. Beyond topological similarity, one can use different biological annotations, such as the Gene Ontology (GO) functional annotation, to compute biologically relevant measures of alignment quality such as GO functional consistency (Aladag and Erten, 2013), defined as the proportion of aligned pairs with more than *k* GO terms in common.

Similar to the use of landmarks in MuNK, IsoRank can incorporate additional similarity information in its computation of the score matrix, so the landmark pairs are provided as a binary information matrix to the IsoRank algorithm. In our experiments, we produce two outputs for method comparison: cross-species pairwise similarity scores and the node-to-node mappings. Thus, in addition to the two measures described above that use the node-to-node mappings, we also evaluated IsoRank and MuNK using AUPR as a measure of enrichment of GO functional consistency with respect to the cross-species pairwise similarity scores. When comparing MuNK to the more recent MAGNA++, MAGNA++ performs very well according to EC (as it optimizes EC directly), but it does not output node scores so we could not directly compare MuNK to MAGNA++ according to AUPR and other metrics. Per the author recommendation, the regularization parameter for the Laplacian in MuNK was fixed at λ = 0.05. Damping can be used in the PageRank step of the IsoRank algorithm, and therefore we performed a grid search with step size 0.05 over possible convexity parameters α ∈ (0,1), optimizing for EC score. As input data, we use the PPI networks for two species of yeast, *S. cerevisiae* and *S. pombe*, extracted from the BioGRID interaction database (Oughtred et al., 2018).

IsoRank performs better on the measures directly related to the node mapping (Table 1). This may be due to the fact that the cross-species similarity coefficients in IsoRank directly incorporate local neighborhood (i.e., topological) information, a fact that the IsoRank greedy algorithm is designed to take advantage of. The MuNK scores predict functional correctness better than the scores produced by IsoRank, suggesting that MuNK’s learned embedded space is biologically meaningful potentially even beyond alignment. In comparing network alignment methods (Guzzi and Milenkovic, 2018) also found that methods that do very well according to the topological quality measures are not very good as far as functional quality is concerned. The interpretability of the embedding space is one of the primary benefits of embedding techniques over standard approaches in the case of network alignment. For example, the embedding space learned by MuNK captures biological information beyond pairwise node alignment, specifically, cross-species synthetic lethal interactions (Fan et al., 2017).

**Table 1:** Comparative analysis of direct vs. embedding methods across a range of problems in network biology.

		IsoRank (α = 0.5)		MuNK (λ = 0.05)	
**A. Network alignment**					
EC		**39.0%**		21.9%	
GOC					
*K* = 20		**63.4%**		57.6%	
*K* = 50		**20.7%**		17.9%	
*K* = 100		**1.2%**		1.0%	
GOC (AUPR)		0.721		**0.746**	
Runtime		26 min 40 s (incl. grid search)		1 min 52 s (incl. alignment)	

		**densityCut** (*K* = 4, α = 0.9)		**Vicus** (*K* = 10, σ = 0.5)

**B. Community detection**					
Buettner (*C* = 2, *C*_t_ = 11)		0.256		**0.316**	
Kolodziejczyk (*C* = 5, *C*_t_ = 4)		0.325		**0.552**	
Pollen (*C* = 13, *C*_t_ = 11)		**0.931**		0.928	
Usoskin (*C* = 9, *C*_t_ = 4)		0.373		**0.591**	
Avg. Runtime		1 min 15 s (incl. parameter grid search)		<5 s	
STRING v9.1 *Homo sapiens*. Included with the Mashup distribution.					

		**GeneMANIA**		**Mashup**	

**C. Function prediction**					
AUPR					
MF		0.327		**0.372**	
BP		0.213		**0.222**	
CC		**0.514**		0.487	
Avg. Runtime		3 min 57 s		14 min 56 s (incl. recommended SVM tuning procedure)	

*Running times were obtained on a 64-bit machine with Intel Core i5-8400 CPU @ 2.80 GHz × 6 with 16 GB RAM running Ubuntu 18.04. Bold refers to the most successful result, according to the referenced metric.*

### Community Detection

One of the natural uses of a network is the identification of clusters, or modules of similar nodes, a task known as community detection (Fortunato, 2010). Community detection methods (Figure 1C) have great uses in biology from protein module identification to disease subnetwork discovery (Ghiassian et al., 2015; Menche et al., 2015). Among the most popular community detection methods on networks are random walk-based approaches including Louvain (Blondel et al., 2008), Infomap (Rosvall and Bergstrom, 2011), Label propagation (Raghavan et al., 2007), and Walktrap (Pons and Latapy, 2005), that came up as best performers in a review comparing these and other approaches (Yang et al., 2016). Originally developed for community detection in social networks, these methods are frequently used in biology (Barabási et al., 2011), for example to identify cancer drivers (Cantini et al., 2015).

Network embedding for the purpose of community detection was covered in a recent review (Hamilton et al., 2017b). The authors hypothesized that due to vector-like embedding representation of a network, there is a wider range of clustering and community detection methods that can be applied to embedded networks as compared to graphs directly. The authors further introduced an encoder-decoder framework that unifies many of the recently popularized approaches, including DeepWalk (Perozzi et al., 2014a) and node2vec (Grover and Leskovec, 2016). A geometric approach, not covered in the review, suggests a scalable embedding of networks in a hyperbolic circle and show that the popular random walk-based community detection methods (Louvain, Infomap, Label propagation, and Walktrap) can be significantly boosted when applied to hyperbolic distances (Muscoloni et al., 2017).

We compared two community detection methods, an embedding-based and a graph-based, on the problem of single-cell RNA-seq (scRNA-seq) analysis. scRNA-seq data has recently emerged as a powerful tool to decipher the heterogeneity of cell populations. This is an important and growing area of network applications where community detection methods are used to perform clustering on the constructed cell-to-cell networks (Wang et al., 2018). Given a gene expression matrix, Gaussian kernel is usually adopted to construct a pairwise similarity network in which nodes represent cells and edge weights depict the similarity between cells.

The first method is Vicus, a generalization of spectral clustering, which we combined with *k*-means clustering in the embedded space. For the network-based approach, we used densityCut, a random walk-based community detection method, which approximates clusters using the density of local neighborhoods. The densityCut method approximates the true network using a *k*-nearest neighbor graph, and selects the number of clusters using an automated procedure. Therefore, this number of clusters was used as input to the *k*-means step of the Vicus evaluation. We used four scRNA-seq datasets, all from *Mus musculus* (Pollen et al., 2014; Buettner et al., 2015; Kolodziejczyk et al., 2015; Usoskin et al., 2015) but which vary according to tissue of origin (neural, blood and stem cells) and have known ground truth labels. We evaluated performance using normalized mutual information (NMI). Vicus outperformed densityCut on three of the four datasets (Table 1).

### Function Prediction

Another fundamental problem in network biology is the inference of protein function from the known functions of its network neighbors (Sharan et al., 2007). The earliest approach to this problem, neighborhood counting (Schwikowski et al., 2000), predicted a protein to be involved in a certain function if a sufficient number of its direct (or up to some specified distance) neighbors had this property. Current state of the art methods are based on similar guilt-by-association principles (Figure 1E). For example, Cao et al. (2013) define a distance metric between proteins that is based on network diffusion, thus capturing similarities that are based on multiple paths in the network.

These single-network methods were generalized in several ways (Cho et al., 2016) integrate information across multiple networks and use a low rank approximation of the network diffusion based similarities to reduce potential noise. The integration challenge is also tackled by (Gligorijevic et al., 2018) who learn a compact node representation using deep autoencoders. In Fan et al. (2017), the cross-species embedding is utilized to infer protein function. Zitnik and Leskovec (2017) suggest a network embedding approach for predicting tissue-specific protein function, which encourages proteins to share features not only with their network neighbors but also with proteins that are active in similar tissues.

Two recent methods were compared on the task of protein function prediction using multiple interaction networks. GeneMANIA performs label diffusion, while Mashup finds an embedding for each of the proteins, allowing one to use traditional classification techniques such as support vector machines (SVMs). The area under the precision-recall curve (AUPR) was used as an evaluation metric. Overall, Mashup performed better with respect to molecular function and biological process annotations, while GeneMANIA performed better on the cellular compartment annotation (Table 1).

### Network Denoising

The application of network biology techniques to experimental data depends on the accuracy and completeness of the network of interest. The challenge of noisy interaction measurements plagues many different types of biological networks, such as Hi-C interaction networks (Rao et al., 2014), cell–cell interaction networks (Wang et al., 2017b), and PPI networks (Saito et al., 2002; Przulj et al., 2004; Chua and Wong, 2008; Higham et al., 2008; Kuchaiev et al., 2009; You et al., 2010; Marras et al., 2011; Alanis-Lobato et al., 2013; Cannistraci et al., 2013; Newman, 2018a,b). Such noise adversely impacts the performance of downstream analysis, calling for methods for network denoising.

The most common approach to denoise any given network is to perform diffusions on the network to exploit high-order structures that can potentially improve the qualities of the direct links between nodes. Diffusion maps (Coifman et al., 2005) employ high-order random walks and then use spectral decomposition to construct an affinity measure. A tensor-based dynamical model (Wang et al., 2012) aims to search high-order paths between pairs of objects through their common nearest neighbors. A low-rank constraint has been employed to help denoise the network manifold (Wang and Tu, 2013). Diffusion-state distance (DSD) (Cao et al., 2013) was utilized to denoise PPI networks and improve the signal-to-noise ratio for better prediction of protein functions. To tackle the problem of transitive edges in networks in a computationally efficient way (Feizi et al., 2013) proposed a simple closed-form solution, called Network Deconvolution (ND), to infer direct links.

An alternative direction of network denoising takes embedding-based approaches. For instance, Mashup (Cho et al., 2016) aims to learn compact low-dimensional vector representation of proteins that best explains their wiring patterns for the input protein–protein association networks by applying a matrix factorization method on the diffused network. The embeddings of the nodes (proteins) reflect the relational structures of the original network, therefore facilitating the downstream applications by feeding the embeddings to a support vector machine.

A recent study (Wang et al., 2018) performed an in-depth comparison between these network denoising methods in three different experimental settings: PPI function predictions, HiC network module detection, and species identification. The study highlighted the advantages of embedding-based methods such as Mashup (Cho et al., 2016) when the network contains distinct cluster structures and the noise level is small. However, it also showed that when the cluster structures are corrupted by high noise, existing methods usually fail to uncover the underlying network structure.

### Pharmacogenomics

Modern pharmaceutical research faces challenges with decreasing productivity in drug development and a persistent gap between therapeutic needs and available treatments (Hodos et al., 2016; Moffat et al., 2017). Network approaches have emerged as a promising direction to address these challenges and improve our understanding of the therapeutic and side effects of drugs (Hopkins, 2008; Berger and Iyengar, 2009). We review three practically important problems within the realm of pharmacogenomics that have been tackled with network embedding methods: drug-target prediction, drug–drug interaction prediction and prediction problems involving small molecules.

Drugs influence biological systems by binding to target proteins and affecting their downstream activity (Imming et al., 2006). Network approaches formulate drug–target interaction prediction as a link prediction task on a graph of drugs/chemicals and the proteins which they interact with (Yildirim et al., 2007; Yamanishi et al., 2010; Perlman et al., 2011; Chen et al., 2012; Cheng et al., 2012; Gönen, 2012; Isik et al., 2015; Zitnik and Zupan, 2016; Luo et al., 2017; Wen et al., 2017; Lee and Nam, 2018). Given such a graph (Crichton et al., 2018) use various node embedding methods, including node2vec (Grover and Leskovec, 2016), DeepWalk (Perozzi et al., 2014b), and LINE (Tang et al., 2015), to embed nodes into a compact vector space in a manner that preserves local network structure. As a result, drugs with many shared target proteins obtain similar embeddings, and vice-versa, proteins targeted by similar drugs obtain similar embeddings. These embeddings are thus well-suited for predicting drug–target interactions by calculating the similarity between embeddings representing the drug and the protein, or by using embeddings as inputs to a machine learning method (Crichton et al., 2018). Alternatively, predictions can be made in an end-to-end fashion, where a neural network learns node embeddings and predicts interactions directly from the graph (Wang and Zeng, 2013; Gao et al., 2018; Wan et al., 2018).

Detecting drug–drug interactions, in which the activity of one drug changes, favorably or unfavorably, if taken with another drug, is an important challenge with significant implications for patient mortality and morbidity (Chan and Giaccia, 2011; Guthrie et al., 2015; Han et al., 2017). Ma et al. (2018) model each drug as a node in a multi-view drug association graph, where edges between drugs in different views encode different types of similarity between drugs. The approach uses graph convolutional networks (Kipf and Welling, 2016) to embed the multi-view graph and attentive mechanisms (Veličković et al., 2018) to fuse information from multiple views and to make learning more interpretable. By such embedding, the approach learns a similarity score between any two drugs and uses the scores to predict drug–drug interactions. While such an approach can be useful to describe drug interactions at the cellular level (Sridhar et al., 2016; Ryu et al., 2018), it cannot predict the safety or side effects of drug combinations. To identify the side effects of drug combinations and provide guidance on the development of new drug therapies (Zitnik et al., 2018) developed an embedding approach that constructs a multi-modal graph of PPIs, drug–protein interactions, and drug–drug interactions, where each drug–drug interaction is labeled by a different edge type signifying the type of the side effect. The approach takes the multi-modal graph and uses graph neural networks as an embedding methodology to distill information about each node’s network neighborhood into an embedding vector without any hand-engineering. The final approach is an end-to-end method for predicting side effects of drug combinations that considers all types of side effects at once. The approach learns embeddings of side effects that are indicative of polypharmacy in patients.

Chemical prediction problems represent another class of practically important graph problems (Ralaivola et al., 2005; Altae-Tran et al., 2017; Gilmer et al., 2017; Gómez-Bombarelli et al., 2018). One key distinction between these problems and standard network prediction tasks discussed above is that chemical prediction problems are graph-level classification problems where individual data examples are graphs (rather than nodes) representing small molecules. Typical prediction tasks aim to predict various molecular properties such as drug efficacy or solubility (Coley et al., 2017; Jin et al., 2017), predict which drugs bind to which target proteins (Morris et al., 2018), and identify sites at which a particular candidate drug binds to a target protein (Feinberg et al., 2018). The input to a predictor is a small molecule, which is commonly represented as a graph in which nodes and edges represent atoms and bonds between atoms, respectively. One difficulty with such inputs is that molecular graphs can be of arbitrary size and shape (Niepert et al., 2016; Xu et al., 2017). However, currently, most machine learning pipelines can only handle inputs of a fixed size. For this reason, state-of-the-art systems use embedding techniques to embed molecular graphs into fixed-dimensional embeddings and then use the learned representations as inputs to a fully connected deep neural network or other standard machine learning methods (Duvenaud et al., 2015; Kearnes et al., 2016). The proposed graph convolution models do not yet consistently outperform traditional structural-based fingerprints, however, their flexibility and potential for further optimization and development have led to models that provide significant boosts in the predictive power over older fingerprints.

## Conclusion

We have reviewed several classes of approaches for network embedding, including spectral-based methods, random-walk based approaches and deep neural network techniques. We have demonstrated the utility of these approaches in a broad set of applications, ranging from network alignment to community detection, protein function prediction, and network denoising. We have also discussed recent embedding approaches in pharmacogenomics. We were interested in seeing whether the field of network embedding indeed enhances the types of questions that can be answered using graph-based approaches and our conclusion is that there is value in both graph-based and graph-embedding-based methods in a variety of applications.

In our experiments we found that depending on the task at hand and metric used, sometimes graph-based methods outperformed network embedding tools. This was the case with, for example, IsoRank beating MuNK with respect to edge conservation in network alignment, whereas MuNK outperformed IsoRank according to the area under the precision recall curve with respect to node mapping. In community detection experiments, our results were reversed, where the embedding method outperformed the graph-based method 3 out of 4 times. In fact, there is no single metric according to which one type of method is consistently better than the other. Even in compute time, where embedding methods outperform graph-based methods most of the time, on the function prediction task graph-based GeneMANIA outperforms the embedding method Mashup. This implies that the choice of graph-based versus embedding-based method will depend on many factors, not just the task at hand, but also the aspect or evaluation measure of highest importance to the user.

The network embedding principles create new opportunities to model large network datasets and move beyond standard prediction tasks of node classification, link prediction, and node clustering. For example, given a partially observed network of interactions between drugs, diseases, and proteins, one might be interested in posing a logical query: “What proteins are likely to be associated with diseases that have both symptoms X and Y?” Such a query requires reasoning about all possible proteins that might be associated with at least two diseases, which, in turn, clinically manifest through symptoms X and Y. Valid answers to such queries correspond to subgraphs. Since edges in the network might be missing because of biotechnological limits and natural variation, naively answering the queries requires enumeration over all possible combinations of diseases (Hamilton et al., 2018) developed a network embedding approach that answers such complex logical queries and achieves a time complexity linear in the size of a query, compared to the exponential complexity required by a naive enumeration-based approach. The approach embeds nodes into a low-dimensional space and represents logical operators as learned geometric operations in this embedding space. They demonstrated the utility of the approach in a study involving a biomedical network of drugs, diseases, proteins, side effects, and protein functions with millions of edges.

We summarize network embedding tools that are used in the biomedical field in Table 2. We expect the importance of these tools to grow with the magnitude and complexity of biomedical data that are being generated.

**Table 2:** A summary of network embedding tools and their applications.

Name of the tool	Availability	What was it applied to
**Denoising**		
Network enhancement	Matlab code http://snap.stanford.edu/ne/	Hi-C interaction networks combining gene interaction networks across tissues
Single-cell representation learning	Binary https://github.com/SuntreeLi/SCRL	Single-cell RNA-seq data
Geometric denoising	http://kuchaev.com/Denoising/	PPI networks
**Network alignment**		
MuNK	Python code and all Anaconda-reproducible experiments https://github.com/lrgr/munk	Cross-species functional PPIs (yeast, mouse, human)
**Community detection**		
Minimum curvilinearity embedding II	https://sites.google.com/site/carlovittoriocannistraci/5-datasets-and-matlab-code/minimum-curvilinearity-ii-april-2012	(i) Cerebrospinal fluid proteomics – neuropathic pain
		(ii) Transcription factor expressions – tissue prediction
Vicus		Single-cell RNA-seq:
		(i) Pollen – neural and stem cells
		(ii) Usoskin – mouse neurons, sensory subtypes
		(iii) Buettner – embryonic stem cells
		(iv) Kolodziejczyk – pluripotent cells
Coalescent embedding	https://github.com/biomedical-cybernetics/coalescent_embedding	Non-biological
**Function prediction**		
Mashup	http://cb.csail.mit.edu/cb/mashup/	Protein function prediction, gene ontology reconstruction, and genetic interaction prediction
OhmNet	http://snap.stanford.edu/ohmnet/	Tissue-specific gene function prediction
Disease gene discovery	http://snap.stanford.edu/pathways/	Disease pathway detection
**Pharmacogenomics**		
Molecular fingerprints	https://github.com/HIPS/neural-fingerprint	Prediction of molecular properties, including drug efficacy, solubility, and photovoltaic efficiency
Decagon	http://snap.stanford.edu/decagon/	(i) Polypharmacy side-effect prediction
		(ii) Drug–drug interaction prediction
Graph convolutional policy network	https://github.com/bowenliu16/rl_graph_generation	Molecular graph generation
Residual LSTM Embeddings	https://github.com/deepchem/deepchem	(i) Drug side-effect prediction
		(ii) Drug toxicity prediction

## Author Contributions

WN did the performance comparisons. All authors participated in writing the manuscript.

## Conflict of Interest Statement

The authors declare that the research was conducted in the absence of any commercial or financial relationships that could be construed as a potential conflict of interest.
